# Effect of Conductive Coatings on Micro-Electro-Discharge Machinability of Aluminum Nitride Ceramic Using On-Machine-Fabricated Microelectrodes

**DOI:** 10.3390/ma12203316

**Published:** 2019-10-11

**Authors:** Asif Rashid, Azat Bilal, Chong Liu, M. P. Jahan, Didier Talamona, Asma Perveen

**Affiliations:** 1 Department of Mechanical & Manufacturing Engineering; Miami University, Oxford, OH 45056, USA; rashidm@miamioh.edu (A.R.); liuc12@miamioh.edu (C.L.); jahanmp@miamioh.edu (M.P.J.); 2 Department of Mechanical & Aerospace Engineering, Nazarbayev University; Nur-Sultan 010000 Republic of Kazakhstan; azat.bilal@nu.edu.kz (A.B.); didier.talamona@nu.edu.kz (D.T.)

**Keywords:** Micro-EDM, ceramics, assistive electrode, silver coating, copper tape, micro-hole

## Abstract

The objective of this study is to investigate the feasibility of machining micro-holes on the non-conductive Aluminum Nitride (AlN) ceramics using micro-electro-discharge machining (EDM) process by exploiting various coating techniques. Although ceramics possess excellent mechanical properties under compressive load condition and superior thermal properties, machining of microscale features on ceramics remains challenging due to the extreme brittleness associated with ceramics. Due to the involvement of higher cutting force and tool wear issue, conventional machining process appears to be unsuitable for machining ceramics. On the other hand, non-contact and negligible process force associated with EDM process makes it one of the competitive processes for machining of ceramics. A series of experiments were carried out on AlN ceramics using “Assistive Electrode” micro-EDM process with a goal of machining blind micro-holes into the ceramics with the aid of on-machine fabricated copper tungsten tools. It was found that multi-layer coatings of silver and copper with copper tungsten electrode resulted in successful machining with high-aspect-ratio holes during powder mixed micro-EDM of AlN ceramics, while micro-holes with less than one aspect ratio are machined without powder addition to the dielectric. It was also observed that comparatively lower level of discharge energies, i.e., lower value of voltages and capacitances were favorable for successful machining of micro-holes in ceramics, even though it results in significantly higher machining time. Despite of relatively low discharge energy usage in micro-EDM, machined surfaces appear to be very rough. The machined surfaces indicate that melting and evaporation, as well as thermal spalling, are the dominating material removal mechanisms. The machined surfaces contained many thermal cracks and porosity on the surface. The elemental composition analysis confirms the presence of aluminum and nitrogen elements on the machined surface. Finally, by careful selection of machining conditions and assistive electrode, successful machining of micro-holes is possible on the non-conductive ceramic surfaces using the micro-EDM process.

## 1. Introduction

Ceramics can withstand temperatures beyond the limits of super alloys, [1] not to mention metal as well as polymer. They can potentially be deployed in very high-temperature regions, which may lead to the increment of operating temperature, output, and efficiency of mechanical components like gas turbines [2]. Enhanced properties of advanced ceramics can offer diverse industrial applications. Machining of these materials has gained greater attention from multidisciplinary researchers due to their versatile properties. Nevertheless, their properties also contribute towards increased difficulty in machining them. Therefore, obtaining exact shapes without generating micro-cracks appears to be a greater challenge yet to be overcome. Conventional machining processes like milling, grinding, drilling, and turning [3] experience bigger challenges due to higher cutting forces and higher tool wear involvement [4], which eventually increases the cost while reducing the productivity. Chemical machining is limited due to their environmental impact. Even with advanced non-conventional machining techniques like laser beam, plasma, and electric beam machining, it seems almost impossible to machine ceramics without generating micro-cracks [5,6,7]

Electrical discharge machining (EDM) has established itself as a viable non-conventional machining process especially for difficult-to-cut materials due to its force free and non-contact nature. It is an electro-thermal non-contact process, which can remove material by melting or vaporizing from any materials that have certain electrical conductivity regardless of hardness. Therefore, complex shapes to materials with high wear resistance can be machined through EDM processes [8,9,10,11]. Structures like bores, grooves, and undercuts in miniaturized hard components are also possible to fabricate through EDM [12,13]. Due to the minimal electrical conductivity requirement by EDM process, machining of non-conductive as well as semi-conductive ceramics using EDM process poses a greater challenge. EDM process has been evolving in order to feed the growing need of non-conductive material machining. Therefore, recent experiments with some sort of assistive method have been carried out where insulators like ceramic materials were machined using assistive processes [14,15,16,17].

**Figure 1:**
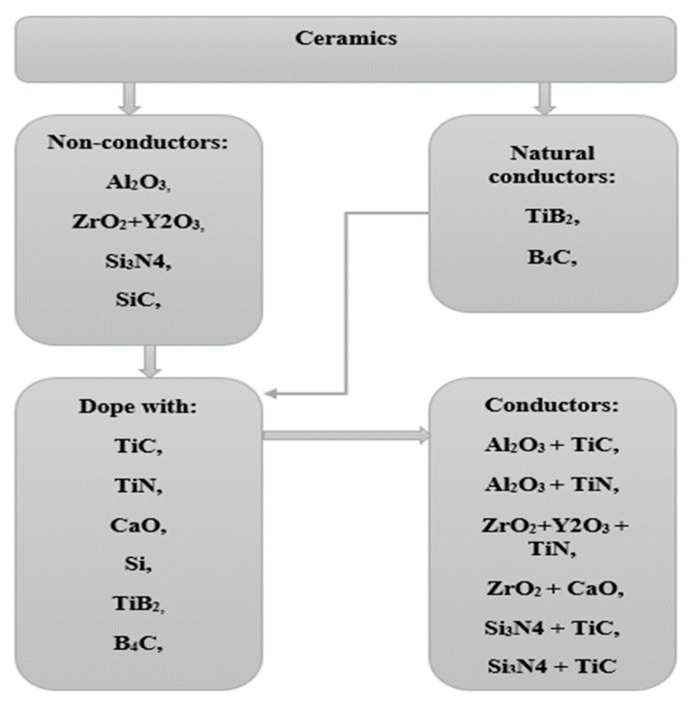
Classification of ceramic materials based on conductivity [18].

As per literature, EDM requires any materials to have electrical conductivity of 10^−2^ Ω^−1^ cm^−1^ for it to be eligible to be machined by EDM. Hence, ceramics like TiN or SiSiC or TiB_2_ can be machined by EDM as they comply with the electrical conductivity requirement. However, insulator ceramics like Aluminum nitride (AlN), Zirconium oxide (ZrO_2_), and Silicon nitride (Si_3_N_4_) do not comply with this conductivity requirement. Three basic mechanisms can be used to generate or enhance electrical conductivity of ceramic materials having conductivity above this value [18]. These are natural electrical conduction by free lattice electrons, doping with conductive materials, and adding impurity atoms into the non-conductive ceramics. Figure 1 shows the commonly used ceramics and the doping materials used to enhance the conductivity of ceramics. Top right block shows the ceramics that are conductive naturally, and top left block represents the non-conductive ceramics which can be made conductive by doping with bottom left block elements. In this way, non-conductive ceramics that can gain certain conductivity are presented by the bottom right block. By enhancing the electrical conductivity of ceramics above critical conductivity required for EDM, machining can be carried out using the EDM/micro-EDM process. Yoo et al. [19] reported one of such cases where SiC was reinforced by Yttrium nitrate. In another study, Kun et al. machined Si_3_N_4_ ceramics using EDM by incorporating TiN. Nevertheless, this incorporation of secondary electrically conductive phase seems to be affecting the mechanical properties; as such, TiN can reduce the fracture toughness, hardness, and flexural strength of ZrO_2_-TiN ceramics [20].

**Figure 2:**
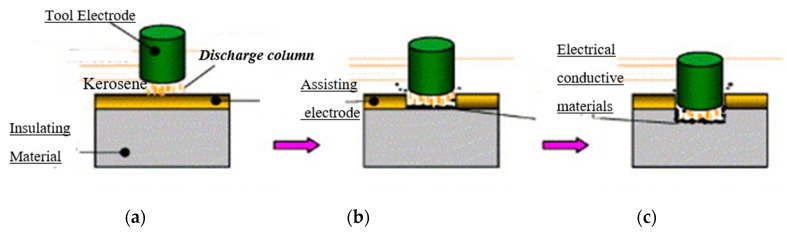
Basic principle of assisting electrode method: (**a**) Discharge for assisting electrode, (**b**) transition from assisting electrode to the insulating material, (**c**) discharge for insulating material [23].

Another method of material removal from ceramics using EDM is the assistive electrode method. Mohri et al. [21] was the first to report on assisting electrode method where a conductive layer is applied on top of non-conductive ceramics. Material removal from ceramic materials during EDM process using assistive electrode method can be characterized by five basic mechanisms (melting and evaporation, thermal spalling, oxidation and decomposition, fusion and vaporization, electromagnetic and electrostatic forces) [17]. This method was the first successful attempt at machining non-conductive ceramics by EDM process. For assisting electrode methodology, EDM process is initiated by a conductive layer on the surface of insulating ceramics, where a carbonaceous oil is used as dielectric. Molecules of the hydrocarbon dielectric oil as well as the workpiece are cracked because of the high temperature generated by the electric discharges. This process assists carbon molecules to bind themselves with ceramics materials. Since these carbon molecules are conductive in nature, discharge may continue to occur even though the conductive layer is removed. As a result, both the conductive layer and ceramics materials beneath that layer will be removed. Any possible conductive coating can be used as assistive electrode. The material removal rate may be improved by adding tool rotation and flushing to the system, as reported in reference [21]. It was later concluded that both the conductive layer and dielectric have an effect on machining ceramics by EDM [22]. Figure 2 shows the material removal mechanism in EDM/micro-EDM of ceramics using “Assistive Electrode” method [23].

As a crucial part of this process is the continuous formation of this intrinsic conductive layer of carbon, it is suggested to investigate the factors that affect its formation such as machining parameters and dielectric [24,25]. It was found that for micro-EDM, the RC type generator circuit is suitable since precise control over energy amount and discharge duration was achieved. Overall material removal rate(MRR) is mainly affected by voltage; however, capacitance and resistance have a significant impact on formation of carbonic conductive layer [26]. Although formation of conductive carbon layer is important, excessive generation of carbonized products is unfavorable since it may lead to unstable machining. Adjustment of current and frequency can be done to decrease the amount of generated carbonized products [27]. Selection of material for tool electrode is also an important aspect. For example, it was found that copper electrode is more suitable for machining with depths smaller than 500 μm, while the tungsten copper shows more stable behavior for machining with depths larger than 1 mm [28]. Regarding the material removal mechanism, it was found that in non-conductive ceramics it is mainly by spalling and that was confirmed by the SEM (Zeiss Supra 35 VP, Zeiss, Germany) images. Moreover, for the stable formation of carbonic conductive layer, there is a lower limit which is 1.2 kVA, and increase in power results in an increase of MRR until 1.4 kVA [29]. In another study, intrinsic conductive layer was investigated during EDM of Si_3_N_4_, SiC, AlN, and ZrO_2_ by using assisting electrode method, and the results suggest that an increase in open circuit voltage leads to an increase in thickness of conductive layer and decrease in bending strength. This implies that slit machining and hole machining of micrometers size on insulating ceramics is possible [30]. The investigation of MRR and recast layer hardness during micro-EDM of zirconia ceramic shows that as gap voltage reduces, MRR increases, and recast layer increases as rotational speed increases since debris materials are not removed completely and therefore they are quenched rapidly [31]. Comparison of machining performance on different non-conductive ceramics was done. Behavior during micro-EDM of ZrO_2_ and Al_3_O_2_ with an addition of secondary conductive phase was observed. According to results, Al_3_O_2_-TiCN has lower surface roughness compared to ZrO_2_-TiN, which is due to higher amount of secondary phase [32]. Another important aspect is the selection of dielectric. In the study of EDM of cobalt-bonded tungsten carbide (WC-Co), three different dielectric fluids, namely, kerosene oil, EDM oil, and distilled water, were used to investigate MRR and TWR (tool wear ratio). It was found that TWR and MRR demand different requirements to be improved since they are opposite in nature, and the one with less harmful effect and consumption compared to dielectric oil needs to be selected [33]. The study related to the machining of Zirconia toughened alumina-titanium carbide (ZTA-TiC), which has high hardness and strength with good ED-machinability and moderate fracture resistance, shows that surface machined by die sinking EDM did not have a glassy or foamy layer, and the bulk material was not damaged [34]. In the study related to machining of Si_3_N_4_ ceramic by EDM, the effect of type of pulses was investigated. It was reported that for relaxation pulses (short pulse duration) machining had higher MRR with leading material removal mechanism of decomposition and oxidation, while for iso-energetic (long pulse duration) it had a better roughness with leading material removal mechanism of melting [35]. For improving performance of EDM for ceramics, addition of conductive powder is helpful. In the study of EDM of reaction-bonded silicon carbide, addition of carbon nanofibers significantly improved machining performance. It was reported that higher carbon nanofiber concentration resulted in better MRR, electro discharge frequency, discharging gap, and decreased tool wear and electrode tip concavity. Since carbon nanofibers disperse discharge energy, several discharge paths are created, and discharge characteristics improve by multiple times [36]. These results agree with another investigation [37] where it was reported that higher concentration of carbon nanofibers results in less electrode wear ratio. This happens because in pure dielectric, ions created from dielectric fluid ionization hit the tool electrode with high energy and momentum.

Literature so far reported on the EDM of non-conductive ceramics process using coating fabricated by CVD of PVD process, which is time consuming and needs costly equipment. In order to reduce the cost of coating fabrication process, several simple coating fabrication techniques have been investigated based on their quality and impact on micro-EDM performance. This study mainly aimed to investigate the feasibility of machining blind micro-holes on AIN ceramics using micro-EDM process with the help of on-machine-fabricated microelectrodes. In this study, some modification on the assistive electrode method was proposed, where deposition of different conductive layers using different methodology on aluminum nitride was exploited to successfully conduct micro-EDM. In addition, the micro-hole characteristics as well as surface topography and modifications were studied as well.

## 2. Experimental Setup, Parameters, and Materials

In this study, all the experiments were conducted on the MIKROTOOLS DT-110 micro-EDM machine. For this study, the micro-EDM drilling option was used. An RC (resistor-capacitor) type pulse generator was used for the micro-EDM process. For the micro-EDM power, the DT-110 micro-EDM machine(Mikrotools, Singapore) has 7 capacitance levels, from 0 to 6, corresponding to the capacitance of 0 pF (stray capacitance), 10 pF, 100 pF, 1000 pF, 10 nF, 100 nF, and 400 nF. The machine has voltage levels from 80 V to 130 V with an increment of 1 V. The machine has the ability of reducing the feed rate automatically, if any short circuit happens. The machine has a resolution feed rate of 0.053 mm/min, which means the minimum feed rate the machine could run is 0.053 mm/min. In order to reduce the runout of the spindles and improve the accuracy of the machining process, microelectrodes were fabricated in situ using the block micro-EDM process. During the machining process, the runout of the spindle was identified first. Then the microelectrodes were fabricated to minimize the runout to zero. Once the diameter of the newly fabricated microelectrode tip is smaller than the minimum runout of the spindle, the old electrode base is still vibrating, but the new electrode tip appears to rotate without vibration or any runout. The electrode fabrication processes have been discussed briefly in the methods section. 

**Figure 3:**
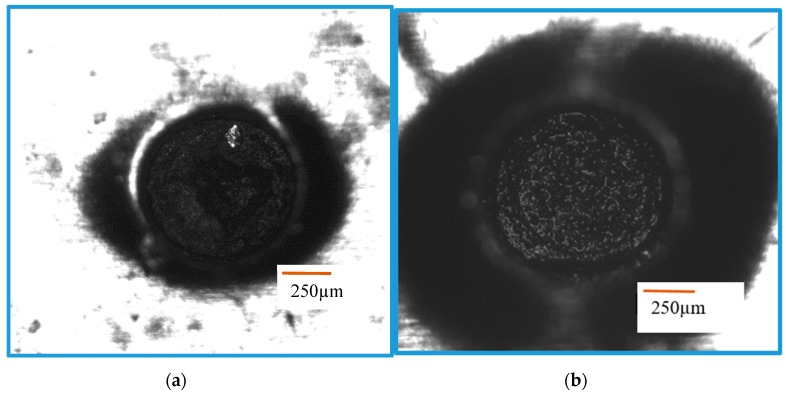

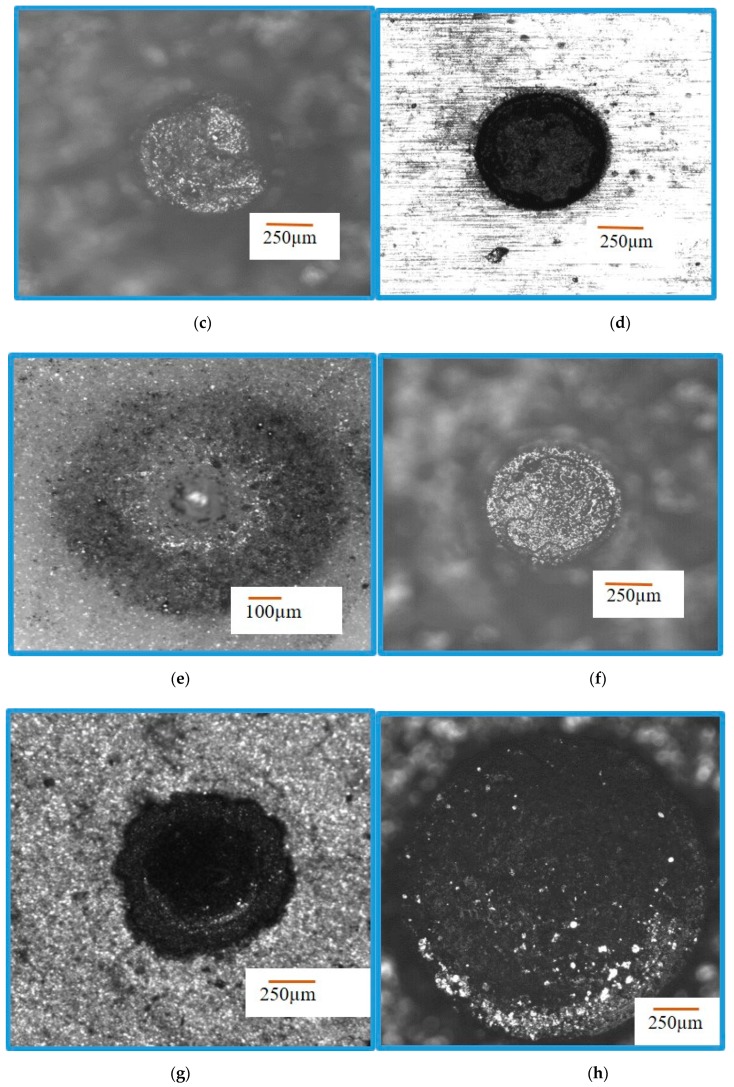

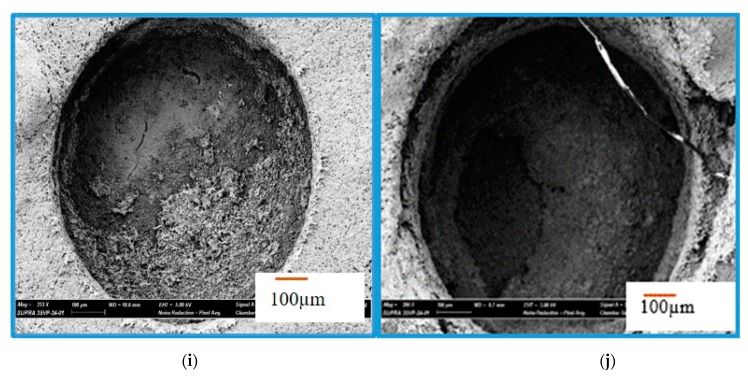
Optical images of few cases of unsuccessful machining. (**a**) Case 25, (**b**) Case 26, (**c**) Case 24, (**d**) Case 21, (**e**) Case 23, (**f**) Case 13, (**g**) Case 8, (**h**) Case 18, (**i**) Case 27 (no machining beyond conductive layer), (**j**) Case 28.

In this study, we have carried out experiments on non-conductive aluminum nitride (AlN) ceramics. For tool electrode materials, different materials were tried, such as tungsten electrode, carbon electrode, and copper tungsten electrode. We have also used different coating materials such as silver paint, copper paint, and carbon paint. Conductive carbon and copper tapes were also considered as coating layer. Silver nanoparticles were used to further enhance the micro-EDM performance. The list of materials, experimental parameters, and machining conditions are listed in Table 1. Table 2 also provides a list for unsuccessful machining and Figure 3 show optical images of some of those unsuccessful machining cases. Among the cases presented in Figure 3, case 21, 23, and 25 demonstrate some machining, whereas the rest of the figures demonstrate no machining with some machining just up to the length of coating thickness.

## 3. Experimental Procedure

### 3.1. In Situ Fabrication of Tungsten Microelectrodes

**Figure 4:**
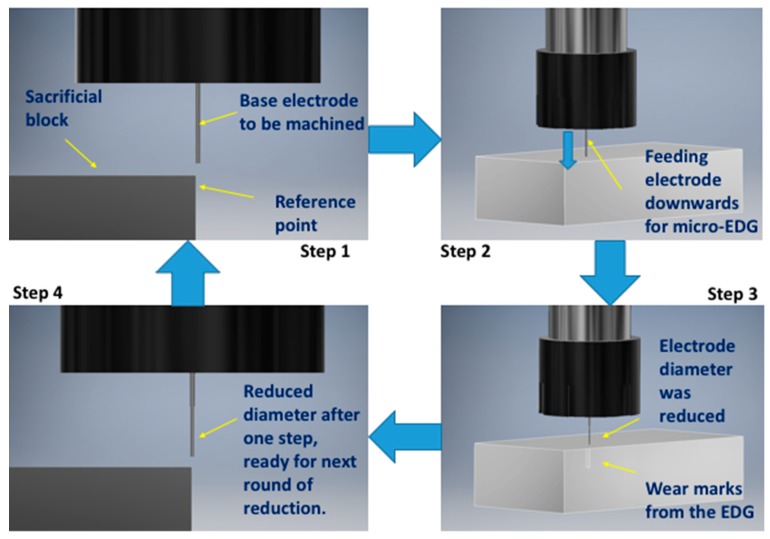
Steps of the microelectrode fabrication using block micro-EDG process (one cycle of reduction of diameter is shown in four steps).

**Figure 5:**
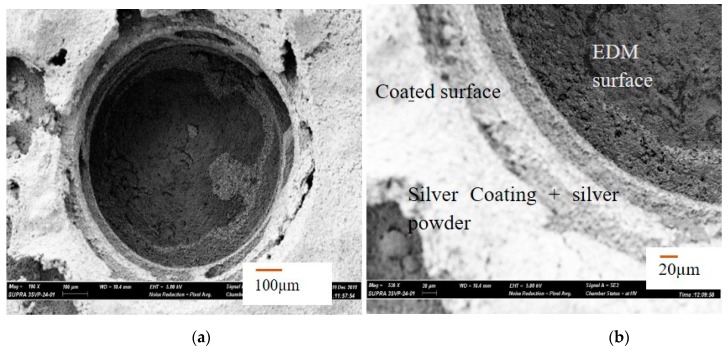
(**a**) SEM image of a micro-hole machined on coated AlN ceramic surface; (**b**) SEM image showing the layers of silver coatings on AIN ceramic surface and the machined surface.

In this study, copper tungsten rods of 600 µm diameter were selected as base electrodes for three successful cases. Tungsten, carbon, and copper tungsten electrodes were used for all successful and unsuccessful attempts. The tool rods were cut to the desired length and machined to the desired diameter by block micro-EDM process. The schematic process of tungsten electrode fabrication is demonstrated in Figure 4. During the electrode fabrication, the electrode was moved to the edge of tungsten block (step 1). Due to the difference in gap distance for different machining conditions, the tool tip was reduced to the desired diameter with several repeated steps. It was found that if a big reduction was intended in one step or the entire microelectrode was targeted in one step, the desired result would not be achieved. In most of the cases, for large reduction in one step, the electrode became tapered, making it unsuitable for use in the machining of blind micro-holes. In most of the cases, five or more steps of reduction were set up for reaching the desired diameter, as shown in Figure 5. The smaller the reduction in each step, the higher the accuracy of the fabricated microelectrodes.

### 3.2. Mechanism of Coatings in “Assistive Electrode Method”

In this study, we have investigated different ways of providing coatings on the ceramic surface for assistive electrode method, as discussed below:

- Coating with a paintbrush: In this process, the conductive coatings of three different materials, silver, copper, and carbon coatings, were provided on the surface of the ceramic workpieces, and then the workpieces were kept in the atmospheric condition overnight for the drying of the coating layer. The process is fully manual and, hence, the surface finish of coating layers is non-uniform and rough. Moreover, the thickness of the coating layers is very difficult to control in this process. Cases 3–8 did not provide successful machining where single copper conductive paint was used. Cases 16 and 18 with single carbon and silver conductive paint also did not experience any machining.
- Coating with conductive tapes: In this process, the conductive tapes of copper and carbon have been applied manually on the surface of the ceramic workpieces. The advantage of this process over paint brush method is that the coating thickness is uniform. However, the major challenge of this technique is that there is some interface gap between the conductive tape and the ceramics surface, which allows air bubbles to trap in between the conductive tape and ceramic surface during EDM. As the assistive electrode method requires the presence of conductive substances on the surface for continuous occurrences of sparks, this method was found to be challenging for obtaining successful machining of micro-holes in ceramics. Cases 9–15 with single layer of copper tape demonstrated no successful machining for different discharge energy level, whereas case 2 with lower energy level (no flushing, no tool rotation) and triple copper layer demonstrated successful machining. Moreover, case 25 demonstrated some machining even with tool rotation of 100 rpm.
- Coating with baking/sintering: One of the major challenges faced during the experiments was to maintain conductive paint/coatings on the ceramics surface, which is integral for the continuation of machining on the ceramics. In order to make strong bonding between the coating layer and ceramic surface, the sintering of the coating was done. In this process, the coating layer was provided using the paint brush manually; after that, another layer of silver nanoparticles was dispersed on top of paint coatings. After that, the sample was baked in a sintering oven for 1 hour at 900 °C. The baking of ceramics resulted in a strong coating of silver and copper coatings on the ceramic surfaces. Case 27 used this type of sample preparation, which resulted in unsuccessful machining when used with tool rotation and flushing.
- Multiple layers of coating with mixed methods: The successful machining of ceramics is dependent on the critical thickness of conductive coating layers. Although the baking of conductive coating paints inside the sintering oven was found to be effective in producing a uniform coating layer, the thickness of the coating layer was not very high. Moreover, if further coating was provided and re-baking was done in the oven, the coating layer could not be increased infinitely. As a result, we provided the first layer of coating by the sintering process with the consecutive layers by manual paintbrush method and re-baking again. In this process, we have tried multiple layers of coating of the same materials only and two time of baking. Case 20 demonstrated no successful machining; however, cases 21 and 23 showed some machining. Case 1 showed successful machining when flushing was not used and low energy level was chosen.

## 4. Results and Discussions

### 4.1. Silver Coating as “Assistive Electrode” 

**Figure 6:**
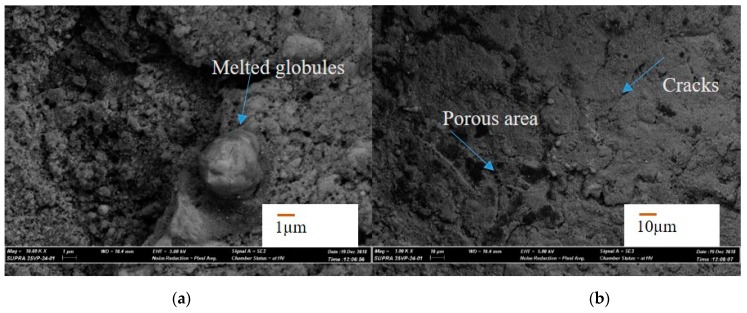
SEM images of the machines surface at the bottom of the blind micro-hole. (**a**) melted globules on the surface; (**b**) thermal cracks and porosity on the surface.

In the first case, a successful machining of hole with 700 µm machining depth using 600 µm diameter tool is presented. Figure 5a shows the SEM image of the top surface of the blind micro-hole machined in AlN ceramics using two layers of silver coating along with silver nanoparticles. The first layer of silver coating along with silver nanoparticles were sintered on the ceramic surface to be machined. The sintering was done in a sintering oven at 900 °C for 60 min. The second layer of silver is then provided on top of the first layer by manual paint brush and sintered in oven at 900 °C for 60 min. Copper tungsten electrode was used for this case. The double layer of conductive coating can be easily observed from the magnified SEM images taken at the edge of the micro-holes, as shown in Figure 5b. There is a clear sign of machining at depths below the two conductive layers of silver coatings. As per the SEM image, machined hole diameter is 759 µm, which gives an overcut of 80 µm approximately for blind micro-hole. Figure 6a,b shows SEM images of the machined surface topography. It can be seen from the images that the machined surfaces are very rough, although the discharge energy used for machining was not very high. The reason behind this higher roughness can be explained with the no tool rotation condition, which let settle down the removed material on the bottom of the hole. SEM image also confirms that material removal mechanism for this ceramic is basically intergranular cracking, which leads to the thermal spalling of whole grains from the machined surface during EDM process. Therefore, thermal composition of ceramics releasing the nitrogen gas as well as thermal spalling act as removal mechanism for this ceramic. The machined surfaces also indicate melting and evaporation, other than thermal spalling as the dominating material removal mechanism. In addition, the machined surface contains many thermal cracks and porosity on the surface, as shown in Figure 6b.

**Figure 7:**
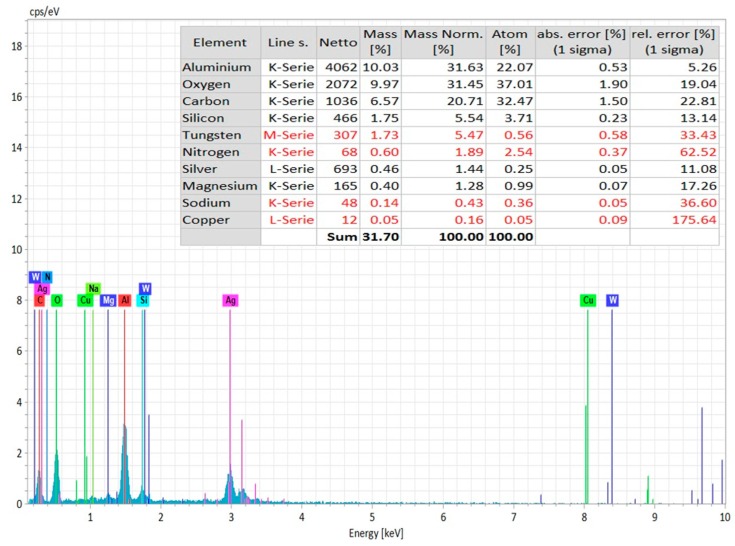
EDS Spectrum analysis on the machined surface (at the bottom of the micro-hole) with elemental composition analysis.

**Figure 8:**
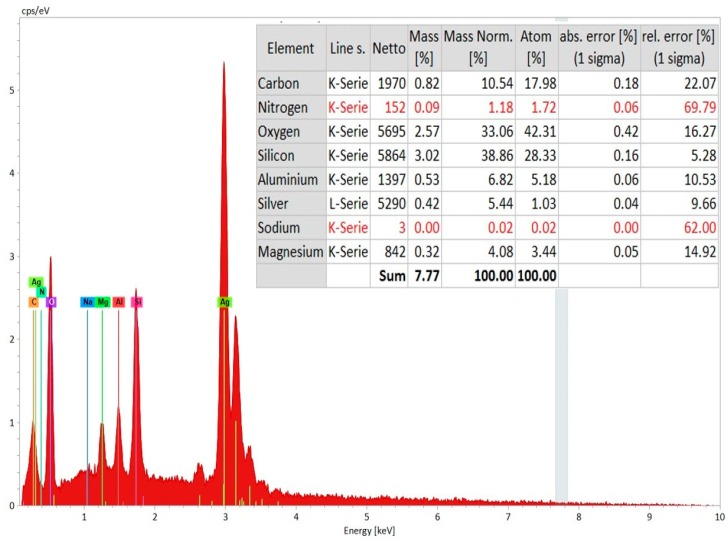
EDS Spectrum analysis on the coating layer (at the top and side of the micro-hole) with elemental composition analysis.

In order to confirm the machining surface from coating layer, EDS spectrum analysis (Zeiss Supra 35 VP, Bruker Espirit 2.0, Zeiss, Germany) was carried out on the top of the hole (coating layer) and the bottom of the hole (machined surface). Figure 7 shows the EDS spectrum analysis of the machined surface that was taken from the bottom of the micro-hole. The elemental composition confirms the presence of aluminum and nitrogen on the machined surface. The source of copper and tungsten present on the surface is the tool electrode. In addition, there is a significant amount of oxygen present on the machined surface, as can be found from Figure 7. Moreover, carbon present on the surface is from the decomposition of dielectric. On the other hand, the EDS spectrum on the top surface of the machined hole (on the layers of silver coating) indicates the presence of silver, oxygen, carbon, and silicon on the coating layer, with no or minimal existence of aluminum and nitrogen, which are the main constituents of the ceramics (Figure 8). 

### 4.2. Copper Coating as “Assistive Electrode”

**Figure 9:**
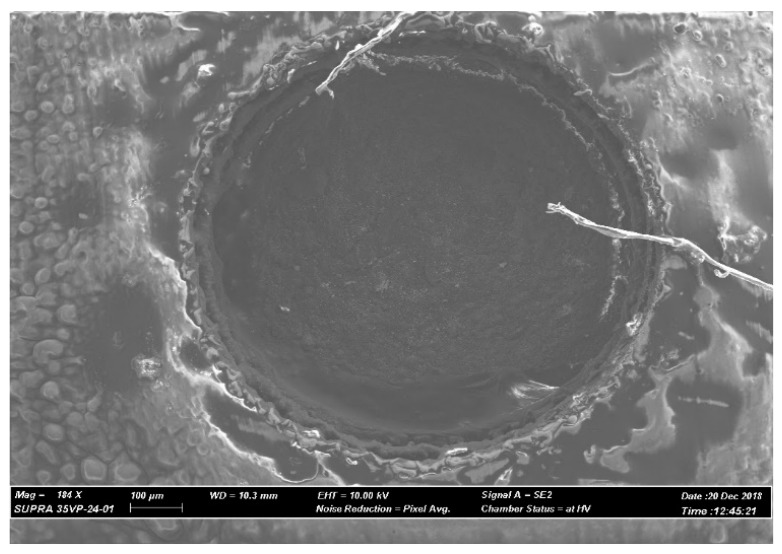
SEM image of the micro-hole on copper tape coated ceramic surface.

**Figure 10:**
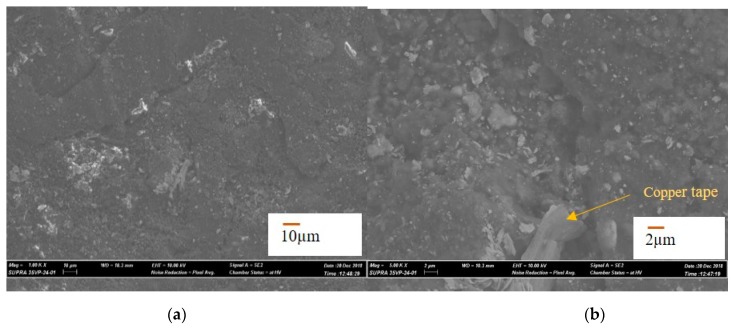
(**a**,**b**) SEM images of the machined surface at the bottom of micro-hole.

Case 2, as shown in Figure 9, shows the SEM images of the machined hole with 150 µm depth and machined surfaces of a micro-hole with triple layered copper tape as assistive electrode. The conditions for machining have been listed in Table 2. The AlN ceramic workpiece was coated with three consecutive layers of conductive copper tape manually. The thickness of 3 layers of conductive copper tape is 150 µm. As dielectric, a 600 µm diameter copper tungsten tool with EDM oil was used. The machining was done without any tool rotation and dielectric flushing to make sure that the conductive craters or particles stay on the machined surface. Figure 9 shows the SEM image of the machined micro-hole. As per the SEM image, machined hole is 730 µm in diameter, which gives overcut of 65 µm approximately. Images of the machined surface at the bottom of the micro-hole is shown in Figure 10. The machined surface was found to be rougher and contain the micro-cracks and debris particles indicating thermal spalling as the dominating material removal mechanism. The SEM image also shows the signs of charging of the copper tape due to the existence of some possible polymeric adhesive substances. There are also signs of deposition of copper tape on the machined surface at the bottom of the micro-hole.

**Figure 11:**
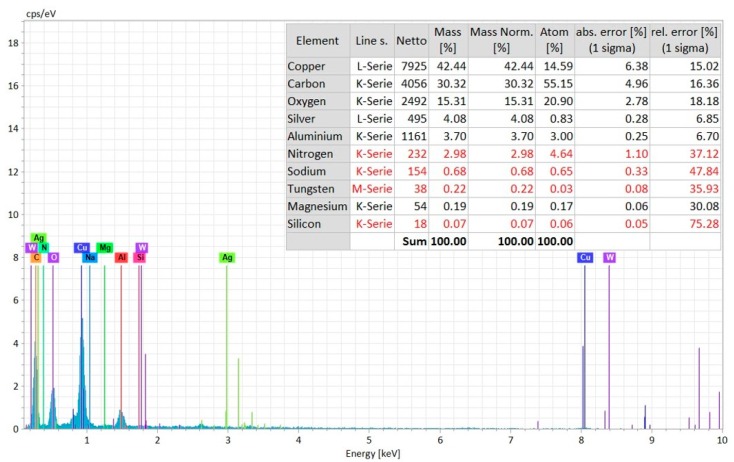
EDS Spectrum analysis of the machined surface (at the bottom of the micro-hole) with elemental composition.

In order to confirm that the machining has been performed successfully beneath the copper tape and proceeded into the AlN ceramics, EDS analysis has been carried out on the copper tape surface and on the machined surface at the bottom of the micro-hole. Figure 11 shows the elemental composition and EDS spectrum analysis done on the machined surface. It can be seen from the spectrum that there is existence of carbon, oxygen, aluminum, nitrogen, and copper on the machined surface. The presence of Al and N confirms that the machining has been carried out on the ceramics surface. The presence of copper indicates that there is some deposition of copper particles from the conductive copper layer. On the other hand, there is some deposition of carbon coming from dielectric decomposition on the machined surface, which is beneficial for the machining to proceed.

**Figure 12:**
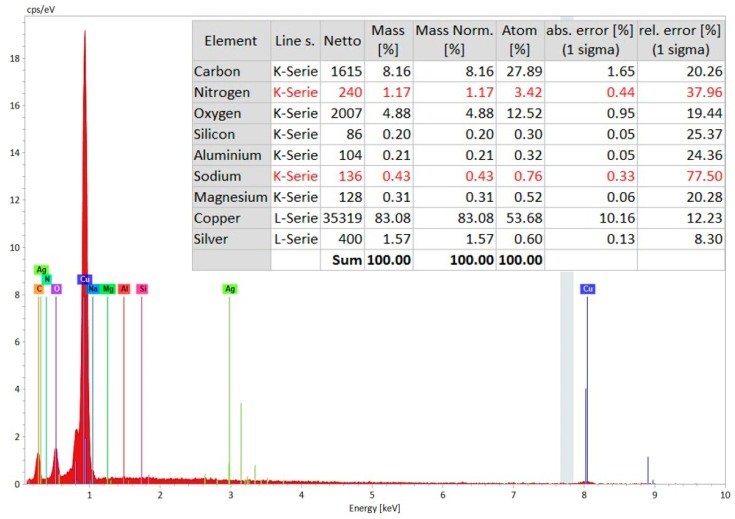
EDS Spectrum analysis of conductive copper tape layers at the area near the top of the micro-hole with elemental composition.

It can be noticed that the major components on the machined surface are carbon, copper, and oxygen with some presence of aluminum and nitrogen. However, the copper percentage is significantly higher, i.e., 83.08, on the conductive copper tape, when the EDS analysis was taken on the un-machined surface of conductive copper tape, as can be seen in Figure 12.

### 4.3. Powder-Mixed EDM with Combined Silver and Copper Coating

**Figure 13:**
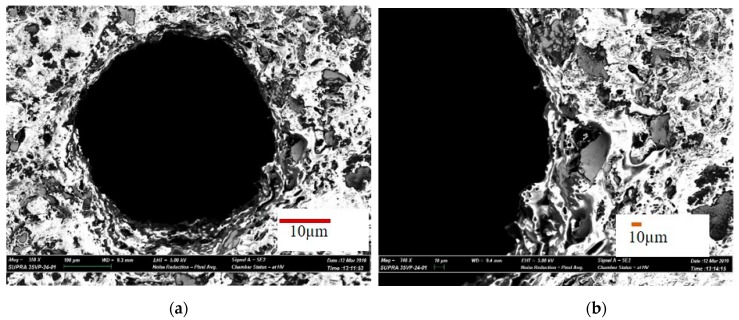
(**a**) SEM image of a micro-hole machined on coated AlN ceramic, (**b**) side wall of the micro-hole surface for powder mixed EDM.

In the third case (Table 2), a successful machining of 450 µm diameter hole with 1693 µm machining depth is presented (aspect ratio close to 4). Figure 13a,b shows the SEM image of the top surface of the blind micro-hole machined on AlN ceramics using two layers of silver coating along with silver nanoparticles. Silver nanoparticles sandwiched between two layers of silver paint along with copper tape on top that were sintered on the ceramics surface to be machined. The sintering was done in a sintering oven at 900 °C for 60 min. Copper tungsten electrode was used for this powder mixed EDM. Copper conductive layer can be easily observed from the magnified SEM images taken at the edge of the micro-holes, as shown in Figure 13b. It is clear from the SEM image that machining depth reaches much below the conductive layers of silver coatings. As per the SEM image, diameters of machined hole and tool are 450 µm and 308 µm, which gives overcut of 71 µm approximately. It can be also seen from the images that the sidewall of machined surfaces appeared to be very wavy compared to the previous two cases, although the discharge energy used for machining was not very high. The reason behind this waviness can be explained by the melted copper tape’s expansion on the hole rim.

**Figure 14:**
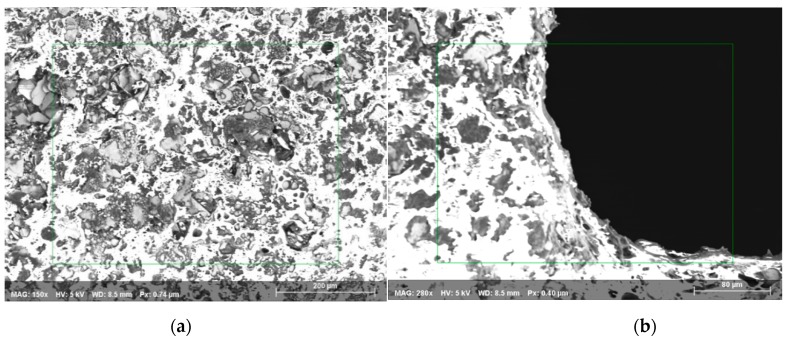
EDS analysis has been taken from (**a**) un-machined coating surface and (**b**) edge of the micro-hole, obtained in AlN ceramic using combined assistive electrode and powder mixed EDM methods (The rectangular box is the area from where EDS spectrum has been taken).

**Figure 15:**
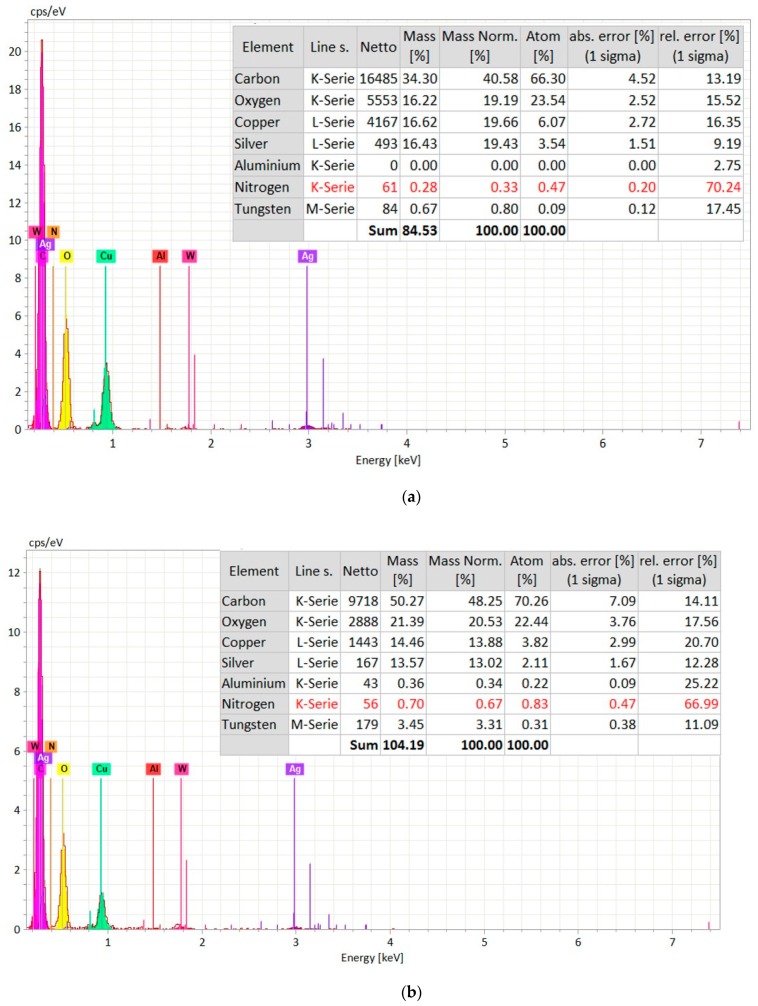
EDS spectrums with elemental composition taken from different spots of the micro-hole machined on AlN ceramic using assisted electrode method with powder mixed EDM; (**a**) un-machined coating surface and (**b**) edge of the micro-hole.

In this case, the depth of the blind hole was too deep to focus using the SEM and, therefore, the EDS analysis at the bottom of the machined surface could not be done. However, the EDS analyses were carried out at three different locations around and on the machined hole. The EDS analyses were carried out on the un-machined coating layer only and at the edge of the micro-hole, as shown in Figure 14a,b, respectively. EDS spectrums were taken at different points of the un-machined and machined surfaces of the micro-hole obtained in AlN ceramics using assistive electrode method combined with powder mixed EDM. The elemental composition for EDS spectrums are shown in Figure 15a,b. It can be seen that the elemental composition of the un-machined surface includes comparatively higher percentage of silver and copper elements due to the coating layer. The percentages of aluminum (Al) and nitrogen (N) are almost zero on the un-machined coating surface. The elemental composition at the edge of the micro-hole is quite different from that of un-machined surface. The percentage of Al and N increased slightly; however, the percentage of carbon (C) and tungsten (W) increased significantly compared to un-machined surface. The slight percentage of Al and N indicates the ceramic elements at the edge of the micro-hole. Whereas, the increased C and W percentage indicates the migration of materials from the tool and the decomposed dielectric into the machined surface, i.e., edge of the micro-hole.

## 5. Conclusions

This study aimed to investigate the feasibility of machining micro-holes on non-conductive ceramics using micro-EDM process. A series of experiments were carried out on AlN ceramics workpieces using various techniques of “Assistive Electrode” method, where several types of coating were deposited on the ceramics surface. The following conclusions can be drawn from this investigation:

- Successful machining of blind micro-holes is feasible on the non-conductive ceramics using this modified assistive electrode method in micro-EDM. However, selection and optimization of machining condition including assistive coating is critical for the successful machining in non-conductive ceramics. It was found that 759 µm diameter holes with 700 µm machining depth could be successfully machined using multi-layer coatings of silver containing silver paints and silver nanopowder with controlled machining environment;
- In this study, consistently successful machining was obtained with silver paint baked on the ceramics surface in the sintering oven. As the coating materials stayed on the ceramics surface during machining process, the sintering of coating provided more consistent machining. Both silver coating and conductive copper tapes were found to produce successful machining of micro-holes on the AlN ceramics. Micro-holes with close to 4 aspect ratio are machined using combined silver and copper tape coatings along with silver nanoparticles mixed EDM. Powder-mixed micro-EDM along with assistive electrode method was found to be more effective than assistive electrode method only, especially in terms of obtaining higher aspect ratios of micro-holes;
- The machining was found to successfully continue without electrode rotation and without flushing, as the deposited carbon layer remains undisturbed and favors further secondary discharging process. In addition, the machining was found to be more stable when the machining was carried out in submerged die-sinking condition rather than side flushing. It was also found that comparatively lower levels of discharge energies, i.e., voltages and capacitances, are favorable for successful machining of micro-holes in ceramics;
- The SEM images of the machined surfaces on the ceramics show very rough surface, although the discharge energy used for machining was not very high. The machined surfaces indicate that melting and evaporation, as well as thermal spalling, are the dominating material removal mechanisms. In addition, the machined surface contains many thermal cracks and porosity on the surface;
- The elemental compositions obtained from EDS analysis confirmed the presence of aluminum and nitrogen on the machined surface. In addition, there was a significant presence of oxygen on the machined surface. The EDS spectrum on the top surface of the machined hole (on the layers of silver coating) indicates the presence of silver, oxygen, carbon, and silicon on the coating layer with no or minimal existence of aluminum and nitrogen, which are the main constituents of the ceramics.

## Figures and Tables

**Table 1:** Experimental conditions, parameters, and materials used in this study.

Parameters	Case 1	Case 2	Case 3
Ceramic	AIN	AIN	AIN
Coating	Baked double layer silver coating with silver nanopowder in between (Both layers baked for 1 h at 900 °C)	Triple layered copper tape. Coating Thickness of 150 µm	Silver nanoparticles sandwiched between two layers of silver with copper on top, baked at 900 °C for 60 min
Tool	Copper–Tungsten	Copper–Tungsten	Copper–Tungsten
Tool diameter	600 µm	600 µm	308 µm
Tool rotation	No rotation	No rotation	No rotation
Dielectric:	Hydrocarbon oil	Hydrocarbon oil	Hydrocarbon oil with Silver nanoparticle (100 mg/L)
Voltage	80 V	100 V	80 V
Capacitance:	10 nF (4)	10 nF (4)	100 nF
Machining time	600 min	120 min	150 min
Depth of hole	700 µm from coating surface	700 µm from the tape surface	1693 µm from coating surface
Flushing condition	No flushing	No flushing	No flushing

**Table 2:** List of machining attempts.

No	Coating	Machining Parameters		Coolant	Spindle Spinning	Tool Material	Machining Condition
		Capacitance	Voltage (V)	Flushing			
1	Baked double layer silver coating with silver nanopowder in between (Both layers baked for 1 h at 900 °C)	10 nF	80	No	No spinning	Copper-Tungsten	Successful machining
2	Copper Tape (triple layer)	10 nF	100	No	No spinning	Copper-Tungsten	Successful machining
3	Copper conductive paint (single layer)	400 nF	130	Yes	1200 RPM	Tungsten	No machining
4	Copper conductive paint (single layer)	100 nF	130	Yes	1200 RPM	Tungsten	No machining
5	Copper conductive paint (single layer)	10 nF	130	Yes	1200 RPM	Tungsten	No machining
6	Copper conductive paint (single layer)	1000 pF	130	Yes	1200 RPM	Tungsten	No machining
7	Copper conductive paint (single layer)	100 pF	130	Yes	1200 RPM	Tungsten	No machining
8	Copper conductive paint (single layer)	10 pF	130	Yes	1200 RPM	Tungsten	No machining
9	Copper Tape (single layer)	400 nF	130	Yes	1200 RPM	Tungsten	No machining
10	Copper Tape (single layer)	100 nF	130	Yes	1200 RPM	Tungsten	No machining
11	Copper Tape (single layer)	10 nF	130	Yes	1200 RPM	Tungsten	No machining
12	Copper Tape (single layer)	1000 pF	130	Yes	1200 RPM	Tungsten	No machining
13	Copper Tape (single layer)	100 pF	130	Yes	1200 RPM	Tungsten	No machining
14	Copper Tape (single layer)	10 pF	130	Yes	1200 RPM	Tungsten	No machining
15	Copper Tape (single layer)	400 nF	130	Yes	1200 RPM	Tungsten	No machining
16	Carbon conductive paint (single layer)	10 nF	80	Yes	1200 RPM	Carbon	No machining
17	Copper Tape (single layer)	100 nF	130	Yes	1200 RPM	Carbon	No machining
18	Silver conductive paint (single layer)	10 nF	130	Yes	1200 RPM	Carbon	No machining
19	Copper conductive paint (single layer)	10 nF	130	Yes	1200 RPM	Carbon	No machining
20	Baked double layer silver coating with silver nanopowder in between (Both layers baked for 1 h at 900 °C)	10 nF	80	Yes	1200 RPM	Copper–Tungsten	No machining
21	Baked double layer silver coating with silver nanopowder in between (Both layers baked for 1 h at 900°C)	400 nF	80	No	No spinning	Copper–Tungsten	Some machining
22	Baked double layer silver coating with silver nanopowder in between (Both layers baked for 1 h at 900 °C) – single layer copper tape on top	10 nF	80	No	No spinning	Copper–Tungsten	No machining
23	Baked double layer silver coating with silver nanopowder in between (Both layers baked for 1 h at 900 °C)	10 nF	80	No	No spinning	Copper–Tungsten	Machining happened at the bottom of the hole
24	Copper Tape (single layer 50 µm)	10 nF	80	No	200 RPM	Copper–Tungsten	No machining
25	Copper Tape (triple layer, 150 µm)	10 nF	100	No	100 RPM	Copper–Tungsten	Some machining
26	Copper Tape (triple layer, 150 µm)	10 nF	130	No	No spinning	Copper–Tungsten	Electrode just repeat going up and down
27	Double layered silver coating with silver nanoparticles in between, baked at 900 °C for 60 min	10nf	80	Yes	1200RPM	Tungsten	No machining
28	Double layered silver coating with silver nanoparticles in between, baked at 900 °C for 60 min	400nf	80	No	No	Tungsten	No machining
